# Methylation at Global LINE-1 Repeats in Human Blood Are Affected by Gender but Not by Age or Natural Hormone Cycles

**DOI:** 10.1371/journal.pone.0016252

**Published:** 2011-01-19

**Authors:** Osman El-Maarri, Maja Walier, Frank Behne, Jan van Üüm, Heike Singer, Amalia Diaz-Lacava, Nicole Nüsgen, Barbara Niemann, Matthias Watzka, Jochen Reinsberg, Hans van der Ven, Thomas Wienker, Birgit Stoffel-Wagner, Rainer Schwaab, Johannes Oldenburg

**Affiliations:** 1 Institute of Experimental Hematology and Transfusion Medicine, University of Bonn, Bonn, Germany; 2 Institute of Medical Biometry, Informatics and Epidemiology (IMBIE), University of Bonn, Bonn, Germany; 3 Department of Gynecological Endocrinology and Reproductive Medicine, University of Bonn, Bonn, Germany; 4 Institute for Clinical Chemistry and Clinical Pharmacology, University of Bonn, Bonn, Germany; University of Miami, United States

## Abstract

Previously, we reported on inter-individual and gender specific variations of LINE-1 methylation in healthy individuals. In this study, we investigated whether this variability could be influenced by age or sex hormones in humans. To this end, we studied LINE-1 methylation in vivo in blood-derived DNA from individuals aged 18 to 64 years and from young healthy females at various hormone levels during the menstrual cycle. Our results show that no significant association with age was observed. However, the previously reported increase of LINE-1 methylation in males was reconfirmed. In females, although no correlation between LINE-1 or Alu methylation and hormone levels was observed, a significant stable individual specific level of methylation was noted. In vitro results largely confirmed these findings, as neither estrogen nor dihydrotestosterone affected LINE-1 or Alu methylation in Hek293T, HUVEC, or MDA-kb2 cell lines. In contrast, a decrease in methylation was observed in estrogen-treated T47-Kbluc cell lines strongly expressing estrogen receptor. The very low expression of estrogen receptor in blood cells could explain the observed insensitivity of methylation at LINE-1 to natural hormonal variations in females. In conclusion, neither natural cycle of hormones nor age has a detectable effect on the LINE-1 methylation in peripheral blood cells, while gender remains an important factor.

## Introduction

DNA methylation is an essential regulatory mechanism in gene expression. In human adult somatic cells, it is present mainly at the 5^th^ carbon position of cytosines in a CpG context, while in iPS and embryonic stem cells, it is present at non-CpG sites [1], [2]. Moreover, two independent groups recently reported the presence of 5-methylhydroxy cytosine in Purkinje neurons and granule cells and in embryonic stem cells in mice [3], [4]. CpG dinucleotides are relatively depleted in the bulk of the genome, but are found in clusters called CpG islands. When present at the promoter of a gene, a highly methylated CpG island may significantly reduce the expression of the downstream gene as is frequently observed in promoter hypermethylation of tumor suppressor genes [5]. CpG sites in repetitive elements, like LINEs and Alus that constitute about 20% and 10% of the human genome, respectively [6], [7], [8], are largely methylated in normal somatic tissue. This is believed to suppress most of their transposition activity [9], [10]. Since these repeats make up about 30% of the human genome the degree of methylation in these repeats will considerably reflect on the average whole genome methylation. At the same time, it has to be taken into account that not all L1 sequences in the genome are full length thus it is to be expected that only a fraction of the Line-1 repeats contain a CpG promoter rich region.

Hence, methylation at LINE-1 repeats is gaining increasing importance as a surrogate marker to distinguish normal from pathological disease tissue. In most carcinogenic tissue hypomethylation of repetitive elements, and particularly LINE-1 elements, is often observed. This includes a wide range of tumors [11], such as prostate adenocarcinomas [12], pancreatic endocrine tumors [13], gastric cancer [14], epithelial ovarian cancer [15], chronic myeloid leukemia [16], uterine cervix [17] and hepatocellular carcinomas [18]. Such LINE-1 hypomethylation was also observed in estrogen-induced rat breast carcinogenesis [19], while hypermethylation was observed during abnormal overgrowth of human placenta, particularly in partial hydatidiform moles [20].

Additionally, LINE-1 methylation (increase as well as decrease) was predicted to be an indicator for the influence of environmental conditions and lifestyle habits on the genome. Exposure to environmental pollutants like exhaust fumes (petrol) was observed to induce hypomethylation at LINE-1 [21]. Baccarelli et al [22] more precisely showed that black carbon particles (<=micromolar 2.5 µM) derived from traffic pollution induce hypomethylation of blood cells LINE-1 sequences. Pilsner et al [23] found a decrease in LINE-1 methylation in cord blood DNA to be associated with maternal exposure to lead. Pavanello et al [24], on the other hand, showed an increase of LINE-1 methylation in association with higher exposure to polycyclic aromatic hydrocarbons (PAHs).

However, before LINE-1 methylation can be used as a marker for pathological diseases or specific environmental exposure, causes of ‘normal/healthy’ variations of LINE-1 in healthy individuals should be thoroughly assessed. Gender, age and hormone influence are some potential affecting factors. Previously, we reported on inter-individual and gender specific variations at LINE-1. Inter-individual variability showed a range of about 5–25% differences, while the gender effect favored a higher methylation in males [25].

In this study, we addressed the effect of age, the natural hormonal variations in vivo during the menstrual cycle and in vitro by treating different cell lines with estrogen, dihydrotestosterone and progesterone. Our results confirmed that age does not significantly affect the global LINE-1 methylation which was also showing significant inter-individual variation but considerable stability during different days of the menstrual cycle over a period of three to four months. Hormone treatment or natural variations had no significant effect on LINE-1 methylation, neither on total peripheral blood cells in women, or on HEK293, HUVEC or MDA-Kb2, but only on T47-Kbluc, which strongly expresses the estrogen receptor. Our results not only highlight the stability (i.e. hormonal and age independence) of LINE-1 methylation in peripheral total blood cells, but also the need to interpret male and female methylation data separately.

## Results

### Age does not have a statistically significant effect on methylation levels at LINE-1 but marginally at F8 locus in females

The first hypothesis in this study was that age is affecting the methylation levels in healthy male or female individuals. For this purpose, we looked at the methylation levels of LINE-1, using a degenerate amplification approach (that unspecifically amplifies different genomic promoter LINE-1 loci; Supplementary Figure S1-A), and at *F8* specific locus in about 300 healthy individuals ranging in age between 18-64 years. None of the studied loci showed a correlation between the measured methylation levels and age of the DNA sample. This was true for all samples collectively, even when we divided the samples into five age groups: 18–20; 21–30, 31–40, 41–50 and 51–64 (Table 1). However, a borderline significance (p value of 0.067) was observed for all collective female samples at *F8* locus CpG-8. The same *F8* locus did not show this tendency at CpG-7, that is 19 bases upstream of CpG-8 (Supplementary Figure S1-C). The correlation with age in females but not in males could be related to the phenomenon of the skewed (and deterioration of) X-chromosome inactivation process associated with aging [26],[27] and its effect on the methylation at the Xq28 where the *F8* is located.

**Table 1:** Methylation at Line-1 and F8 and its correlation with gender and age.

Region		Male						Female					Gender Dif.	
						correlation with age					correlation with age		Ave. Dif.	t-test (p)
		Age	Av. %	SD	n	r	p	Av. %	SD	n	r	p		
**F8**	**CpG7**	**18–20**	**81.77**	3.10	16	−0.0857	0.7522	**78.77**	4.31	17	−0.2774	0.2811	**3.00**	**0.02919**
		**21–30**	**82.57**	3.59	73	0.1084	0.3609	**78.79**	3.55	76	0.0039	0.9733	**3.77**	**0.00000**
		**31–40**	**82.13**	3.52	31	0.1491	0.4235	**78.95**	4.34	29	−0.2488	0.185	**3.18**	**0.00258**
		**41–50**	**83.06**	2.77	14	0.5015	0.0677	**80.27**	3.39	23	−0.1289	0.5578	**2.78**	**0.01400**
		**51–64**	**81.24**	3.06	19	−0.0424	0.8631	**78.95**	2.55	10	−0.0083	0.9819	**2.29**	**0.05351**
		**All**	**82.25**	3.38	153	−0.0444	0.5854	**79.11**	3.72	157	0.0649	0.4197	**3.14**	**2.96E-14**
	**CpG8**	**18–20**	**69.29**	2.96	16	−0.4584	0.0741	**56.88**	5.79	17	−0.437	0.8679	**12.41**	**1.17E-08**
		**21–30**	**68.79**	4.33	73	−0.0194	0.8708	**59.41**	4.77	76	0.0464	0.6885	**9.37**	**3.71E-25**
		**31–40**	**69.40**	6.46	31	−0.1855	0.3177	**57.96**	5.82	29	0.0238	0.9008	**11.44**	**1.00E-09**
		**41–50**	**67.86**	6.52	14	−0.1351	0.6452	**60.42**	6.10	23	0.1324	0.5471	**7.44**	**0.00126**
		**51–64**	**68.01**	5.24	19	−0.1235	0.6144	**61.41**	5.43	10	0.0709	0.8456	**6.60**	**0.00361**
		**All**	**68.76**	5.10	153	−0.0845	0.2991	**59.18**	5.49	157	0.1462	0.0677	**9.58**	**1.87E-43**
**L1**	**CpG15**	**18–20**	**40.68**	4.57	15	−0.3883	0.1526	**39.24**	3.16	15	−0.1189	0.6729	**1.45**	**0.32157**
		**21–30**	**39.60**	2.85	67	−0.1117	0.3679	**38.61**	4.09	78	0.1091	0.3384	**0.99**	**0.09856**
		**31–40**	**38.80**	2.85	30	−0.0118	0.9506	**38.64**	3.28	33	−0.0322	0.8588	**0.16**	**0.83534**
		**41–50**	**39.03**	3.55	17	−0.3871	0.1247	**38.23**	3.48	21	−0.489	0.0245	**0.79**	**0.49372**
		**51–64**	**39.89**	2.43	20	0.3407	0.1415	**37.89**	2.16	11	−0.2965	0.3758	**2.01**	**0.02975**
		**All**	**39.49**	3.08	149	−0.0531	0.5205	**38.54**	3.55	159	−0.0764	0.3383	**0.94**	**0.01493**

The samples were analyzed in 5 groups of 10 years intervals except for the first group and collectively in all samples.

SD: standard deviation; n: number of individuals; r: Pearson correlation coefficient.

### Gender-specific methylation levels at global LINE-1 and F8 locus

Previously [25], we have shown that in 24-year old healthy males and females, a significant association between LINE-1 methylation and gender exists: males were higher methylated. In order to follow this male-female difference in different age groups we analyzed the data generated in this study for a gender effect. A 0.94% higher methylation in males was observed for all combined age groups (t-test p = 0.014) (Table 1). When dividing all samples into five different age groups (18–20, 21–30, 31–40, 41–50 and 51–64) males were higher methylated in all age groups, however, significant difference was only observed in the age group of 51–64 (p value 0.029). The lack of significance in other groups could be attributed to the relatively smaller sample size as compared to all groups combined.

As control, we also investigated two X-linked specific CpG sites in exon 14 of the *F8* gene that we had reported earlier to show a clear difference between males and females, with the latter being less methylated [25]. In this F8 case, the lower methylation in females could be explained by the general hypomethylation observed at the inactive female X-chromosome and the hypomethylation in the body of unexpressed genes on the inactive X [28], [29]. Here again and also at the two studied CpG sites (in the *F8* locus), we could clearly see significant differences in all age groups combined as well as in the individual age groups (apart from a border line significance at CpG7 for age group 51–64 (p = 0.053). (Table 1).

We observed a relative difference between male and female methylation when comparing our previous study [25] with this one (previous study = 3.46%; this study = 0.94%). This is due to the fact that different bisulfite treatment protocols were used (agarose bead method [30] vs. EpiTect bisulfite kit from Qiagen) and to differences in annealing temperature influencing the average methylation levels when using a degenerate primer amplification approach (Supplementary Figure S2). In addition, the average age in the first study was 24 years, while in this study we were analyzing samples ranging in age between 18 and 64 years.

### LINE-1 and Alu methylation is stable during different stages of the menstrual cycle regardless of hormone levels

The influence of estradiol, progesterone and testosterone levels on the methylation levels at LINE-1 (SN-8 (CpG15) and SN-9 (Supplementary figure S1-A)) and Alu (SN-1 and SN-4 (Supplementary figure S1-B)) in healthy females during different stages of their menstrual cycle was tested using a mixed model. No significant dependence of the methylation values on any one of the three measured hormone levels was observed (p value 0.17-0.95) (Table 2). As control, we tested the dependency of methylation at CpGs within one studied locus (i.e.: LINE-1 SN-8 and SN-9; Alu SN-1 and SN-4). Some dependency is expected as the two CpGs which are amplified in one reaction and are close to each other, tend to have correlated or dependent methylation levels [25]. Indeed, both CpGs within LINE-1 and within Alu show a dependency on each other (within one locus) (p value for LINE-1 SN8 and SN9: 5E-04; p value for Alu SN1 and SN4: 1.25E-05). However, no dependency between any one of the two CpGs in LINE-1 and any one of the two CpGs in Alu was observed. This is in contradiction to previously published data including our own [25], possibly due to the relatively small number of independent measurements of only 17 cases. In total, there were 165 samples where LINE-1 and Alu methylation at 2 CpG sites at each locus was measured. However, these were derived from only 17 independent individuals. Estradiol and progesterone are also expected to show a correlation since during the menstruation phase, both hormones are very low, while in the proliferative phase as well as in the secretory phase, both have substantially higher values. This dependency was observed as expected (p value for estradiol and progesterone: 0.0032).

**Table 2:** The influence of different hormone levels as fixed and women and month of cycle as random effects on different methylation measurements calculated by a mixed linear model is shown in this table.

Effect	dependent	DenDF	FValue	ProbF
**Estradiol**	**L1_SN9**	103	1.17	0.2820
**Estradiol**	**L1_SN8**	103	0.01	0.9086
**Estradiol**	**ALU_SN4**	95	0.82	0.3669
**Estradiol**	**ALU_SN1**	105	1.00	0.3208
**Estradiol**	**Progesterone**	94	9.14	**0.0032**
**Estradiol**	**Testosterone**	83	0.66	0.4194
**Progesterone**	**L1_SN9**	92	1.92	0.1697
**Progesterone**	**L1_SN8**	92	0.01	0.9149
**Progesterone**	**ALU_SN4**	84	0.52	0.4732
**Progesterone**	**ALU_SN1**	94	0.02	0.8861
**Progesterone**	**Testosterone**	76	1.46	0.2308
**Testosterone**	**L1_SN9**	81	0.00	0.9500
**Testosterone**	**L1_SN8**	81	0.53	0.4679
**Testosterone**	**ALU_SN4**	77	0.87	0.3546
**Testosterone**	**ALU_SN1**	82	0.81	0.3721
**L1_SN8**	**L1_SN9**	112	13.03	**0.0005**
**L1_SN8**	**ALU_SN4**	102	0.58	0.4480
**L1_SN8**	**ALU_SN1**	112	0.81	0.3705
**L1_SN9**	**ALU_SN4**	102	0.38	0.5401
**L1_SN9**	**ALU_SN1**	112	0.05	0.8293
**Alu_SN1**	**ALU_SN4**	104	21.06	**1.25E-05**

The dependence of hormone level on each other's and the dependence of methylation at each locus on other loci are also shown. The probability of <0.05 is considered as significant. (DenDF: Denominator degrees of freedom; FValue: Quantile of the F distribution; ProbF: P value of the F distribution).

### Inter-individual variations in LINE-1 and Alu methylation are stable over 3-4 months donation period

**Figure 1:**
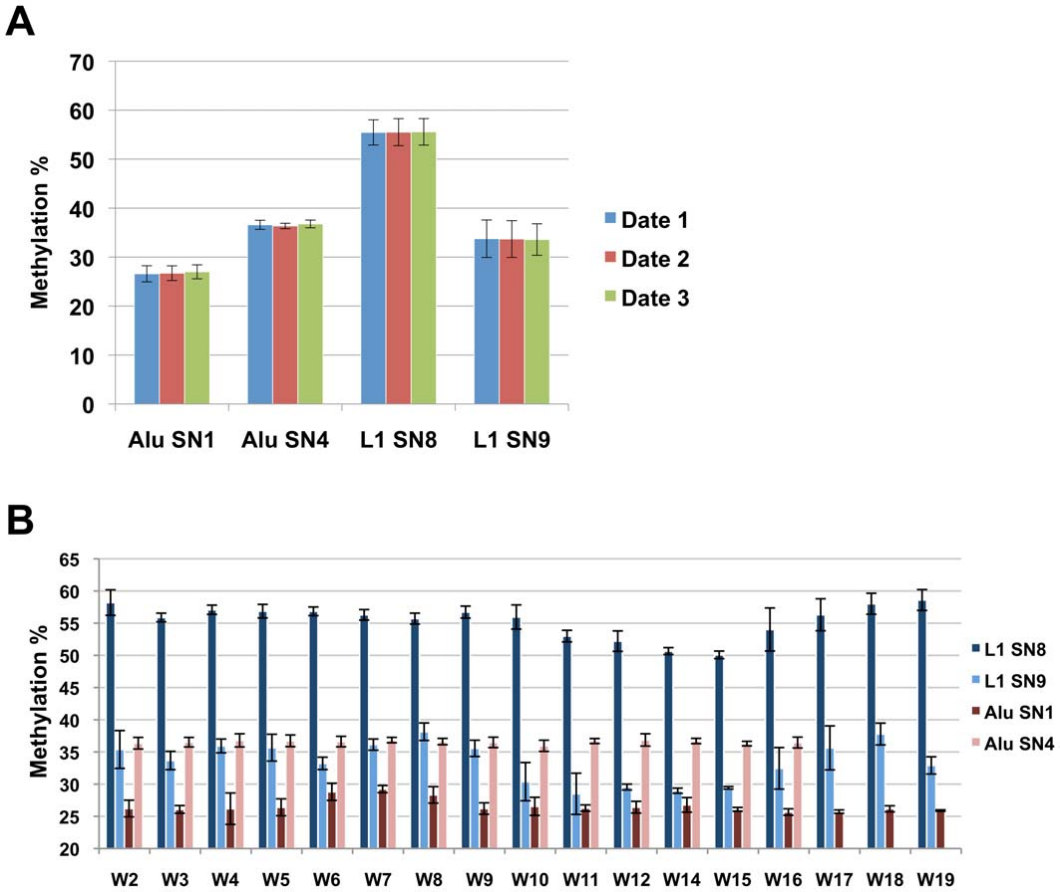
Average methylation levels at LINE-1 (SN9 and SN8) and Alus (SN1 and SN4) A) on three dates during the menstrual cycle (Date 1: during the menstruation phase, 55 samples; Date 2: near the ovulation phase, 56 samples; Date 3: during middle of secretory phase, 53 samples). The data are the averages of all participating women and all measurements. B) of all blood samples taken from a given female donor. The graph shows the inter-individual variability between donors that is persistent over 3–4 months period. The donor number is given on the horizontal axis.

Next, we wanted to test the stability of the measured methylation values over time within one woman and between different women. For this purpose, we used an ANOVA variable model approach. The variability parameter within one woman was shown to be much lower (range between 0.61 and 4.01) than between different women (range between 0.6 and 94.34) (Table 3). Variability between women at LINE-1 was much more pronounced than at Alu, with the latter being particularly stable at Alu SN-4 (Table 3; Figure 1).

**Table 3:** The stability of the methylation measurement at Line-1 and Alu both within (estimated by mean square of the residuals: Var_Error) and between women (estimated by mean square of the model: Var_Model) was calculated using the ANOVA approach.

	Model (Variability between women)					Error (Variability within women)				
Dependent	DF	Var	Cl_1	STD	Cl_2	DF	Var	Cl_1	STD	Cl_2
**L1_SN9**	16	94.34	7.23	9.71	14.78	147	4.02	1.80	2.00	2.26
**L1_SN8**	16	52.83	5.41	7.27	11.06	147	2.13	1.31	1.46	1.65
**Alu_SN4**	14	0.65	0.59	0.81	1.27	135	0.61	0.70	0.78	0.89
**Alu_SN1**	16	12.09	2.59	3.48	5.29	149	1.29	1.02	1.14	1.28

Upper and lower confidence limits are also shown. (DF: degree of freedom, Var: calculated variability parameter, Cl_1 and 2: Confidence limits of the standard deviation, STD: standard deviation).

### Hormone treatment of different cell lines

#### RT-PCRs and western blot of hormone receptors

**Figure 2:**
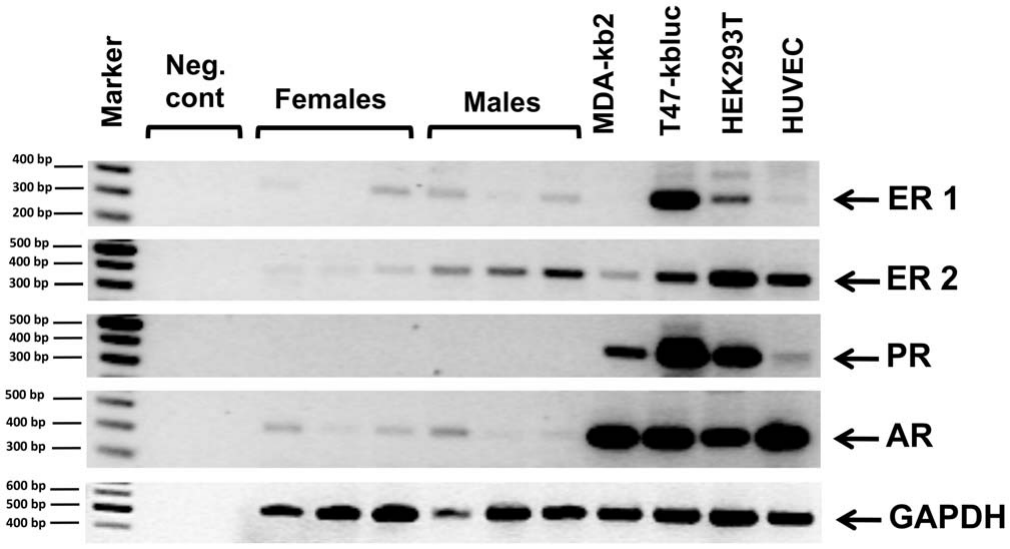
RT-PCR of the receptors in MDA-Kb2, T47-Kbluc, HEK293T, HUVEC cells and male and female peripheral blood derived cells. Estrogen Receptor 1 (ER-1 (alpha)), Estrogen Receptor 2 (ER-2 (beta)), Progesteron Receptor (PR), and Androgen receptor (AR).

Qualitative RT-PCRs showed that estrogen receptor-1 is strongly expressed in T47D-Kbluc, moderately in Hek293T, slightly in HUVEC and male and female peripheral blood cells. Estrogen receptor 2 is moderately expressed in all cell lines, relatively weaker expressed in male and even weaker in female peripheral blood cells. Progesterone receptor is slightly expressed in HUVEC, moderately in Hek293T and MDA-kb2 cells and strongly in T47D-Kb2 cells but not detectable in peripheral blood cells. Lastly, androgen receptor is strongly expressed in Hek293T, MDA-kb2 and in T47D-Kbluc and slightly in male and female peripheral blood cells (Figure 2). Western blots against ER and AR largely confirmed the RT-PCR results and showed that the ER protein (within the sensitivity of western blot) is not detectable in peripheral blood cells but present in T47D-Kb2 cell line (data not shown). The androgen receptor on the other hand, was detected by western blot only in T47D-Kb2 and MDA-Kb2 cell lines, again confirming the RT-PCR results. The primers used for the RT-PCRs are listed in Supplementary Table S1.

#### Methylation levels at LINE-1 and Alu in T47D-KBluc are affected by estradiol but not dihydrotestosterone

**Figure 3:**
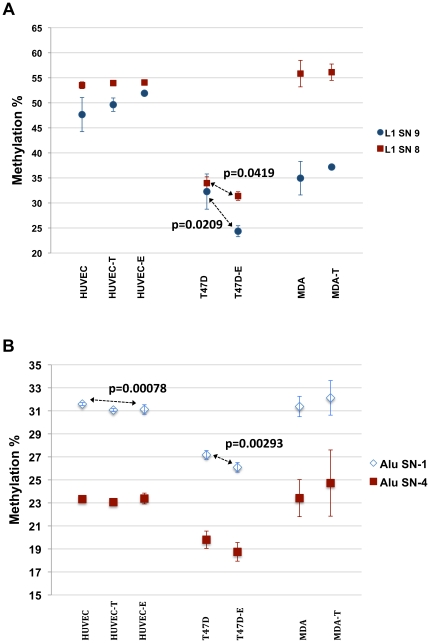
Effect of Hormone treatment on DNA methylation levels at A) LINE-1 and B) Alu. The 48 hours hormone response in HUVEC, T47D-KBluc and MDA cells. T and E stands for dihydrotestosterone and estradiol respectively. Each value corresponds to three different cell culture treatments, each measured thrice for methylation (making a total of 9 measurements).

Sex hormones could be a contributing factor to the gender difference in methylation at least within repetitive elements. To test this we treated HUVEC (primary endothelial cells) and T47D-KBluc cell line with E2 (100 nM final concentration corresponding to about 68-fold the upper normal physiological levels of 400 pg/ml in healthy females) and HUVEC cells and MDA-Kb2 with DHT (100 nM final concentration corresponding to 36-fold the upper normal physiological levels of 0.8 ng/ml in healthy females) for 48 hours. Next, we analyzed methylation levels at Alu and LINE-1 repeats (with degenerate primers). No difference was observed before and after treatment or within controls for the DHT treatment. E2 treatment resulted in a slight but significant drop in methylation in T47D-KBluc at both LINE-1 CpGs and at Alu CpG1. A slight but statistically significant drop in Alu methylation of CpG-1 in E2-treated HUVEC was also observed (Figure 3).

#### Treatment of HEK293 cells with hormones over an extended period of four weeks did not alter the DNA methylation at LINE-1 elements

As we cannot specify the time lapse required between hormone treatment and change in DNA methylation levels or patterns we treated HEK293 cells with different hormones for an extended period of four weeks. LINE-1 methylation was analyzed at one-week intervals. No difference in level of methylation was observed (data not shown).

## Discussion

In this study, we addressed parameters that could possibly contribute to the variability of methylation, mainly at LINE-1 repetitive regions. The stability over a 3–4 months period, the inter-individual variations, and the effects of age, gender, and sex hormones were thoroughly investigated. All of these parameters, with the exception of gender, were seen to not affect levels of DNA methylation in peripheral blood cells as inferred from methylation of repetitive DNA. However, estrogen was an exception as it induced hypomethylation of LINE-1 in an in-vitro system in T47-Kb2 carcinogenic cell line that strongly expresses the ER-alpha as detectable by RT-PCR.

Our data show that some degree of variability exists at *F8* single loci and at LINE-1 and Alu repeats. The two CpG sites in the F8 locus were the most variable (SD ranging between 3.38 and 5.49). This is consistent with our previous study [25] and confirms earlier reports that used similar sensitive quantitative methods to investigate the variability of methylation at several single loci [31], [32]. Our data also confirm the lack of significant correlation between LINE-1 methylation and age and the stability over time (3–4 months period) that has been reported by others [11], [33], [34], [35], [36]. Several studies have investigated the effect of age on LINE-1 methylation. However, none found a statistically significant effect. Nevertheless, Bollati et al and Iacopetta et al, described a decrease tendency associated with age (summarized in Supplementary Table S2) [11], [33], [34], [35], [37], [38].

Others and we previously reported gender difference at LINE-1, which was also confirmed in this study [25], [37]. On the other hand, several other studies [11], [34], [35], [38] that investigated methylation at global LINE-1 failed to detect such global differences in methylation between males and females (these studies are summarized in Supplementary Table S2). This could be due to several factors: 1) different detection methods, sensitivity of the method, different tissues studied and size and age of the studied samples; 2) specificity of the primers and whether they selectively amplify the equally methylated regions in both genders or target differentially methylated X-Linked regions; 3) annealing temperature used to amplify the genome-wide dispersed LINE-1 elements. Indeed, we found a clear correlation between annealing temperature and differences in methylation between males and females with more pronounced differences observed at higher temperatures (Supplementary Figure S4). This is probably due to the specificity of amplification of more gender-specific LINE-1 differentially methylated regions at higher temperatures.

However, what are these gender-specific differentially methylated regions and where are they located in the genome? The main source we could think of is the X-chromosome inactivation that is characterized by a hypomethylated inactive X-chromosome. This has been confirmed for single copy genes on the inactive X [29]–[30] but the methylation status of specific LINE-1 sequences has not been directly studied to date. However, from the data presented in this study we cannot inferred if this observed female Line-1 hypomethylation could be originated solely from the inactivated X or whether autosomal regions would also contribute to this phenomenon. Detailed future study of methylation of autosomal and X-linked specific Line-1 regions are necessary to further clarify this phenomenon (this is currently being addressed by our group).

Once we had reconfirmed, using a large sample cohort, that gender is an important factor to predict LINE-1 methylation, we investigated whether sex hormones also contribute to this effect in addition to the process of X-chromosome inactivation, i.e. estrogen could be responsible for the decrease in methylation females or testosterone could increase the methylation in males. Although several studies addressed the effect of 17 β-estradiol, none could establish a universal link between estrogen and methylation levels. Aniagu et al found a hypomethylation effect of E2 in HepG2 cell lines but not in primary human hepatocytes [39]. Kovalchuck et al detected a loss of global methylation and LINE-1 hypomethylation in induced mammary carcinomas in estrogen treated female August Copenhagen Irish rats [19]. Feng et al, [40] on the other hand, found a strong association between the promoter methylation status of several genes and the expression patterns of estrogen and progesterone receptor genes. Hence, the effect of estrogen on methylation most likely depends on cell type, tissue and DNA sequence.

Hereon we addressed the specific question of whether hormones affect methylation of LINE-1 and Alu repeats. We investigated this using two sets of samples. First, an in vitro cell culture system using human cell lines (HEK293, HUVEC, T47D-KBluc, MDA-kb2) that have defined but different expression patterns of androgen, progesterone and estrogen receptors. These cell lines were treated with estrogen, progesterone or DHT. The second set is from 17 healthy female blood donors where three blood samples were withdrawn during each menstrual cycle (for 3–4 consecutive months) during the menstruation phase, at the end of the proliferative phase and at the middle of the secretory phase. The peripheral blood cells from which DNA is extracted are collectively called leukocytes. These include different subgroups that differ in lifespan and percentages among total leukocytes. Thus we distinguish neutrophils, lymphocytes, monocytes, eosinophils and basophiles that make up (on average) 55.5%, 35%, 8.6%, 3.5% and 0.75%, respectively. For most of them, lifespan in the blood stream is still not very well defined although the literature suggests a lifespan of 25 hours to 5.4 days for neutrophils, several weeks to years for lymphocytes, 1–3 days for monocytes, 26 hours for eosinophils, and 1–3 days for basophiles [41], [42], [43], [44], [45]. Therefore, about 65% of the nucleated peripheral blood cells are relatively short lived and would be subject to monthly hormonal fluctuations during the female menstrual cycle.

In both samples sets, no link could be established between either of the hormones and methylation levels at different repeat regions (Alu and LINE-1) with the exception of a slight but significant hypomethylation effect of estrogen on the T47D-Kbluc cells. The sensitivity of the latter cell line to estrogen could be explained by the higher expression of estrogen receptors (Figure 2) and the presence of ER-alpha in this cell line, but not in the other cell lines or the peripheral blood-derived cells as seen by western blot (data not shown). Therefore, the estrogen receptor may mediate the hypomethylation effect by either lowering the expression of a factor needed for methylation or inducing the expression of a de-methylating factor. We also cannot exclude that this specific cell line is particularly more prone to such an estrogen effect due to the fact that it is a mammary carcinoma cell line that could have multiple cellular transformations making it vulnerable to an estrogen effect. This is in agreement with the fact that sex hormones have different epigenetic effects on male and female brain development, which could be induced by differences in promoter methylation and expression of estrogen and progesterone receptor [46]. On the other hand the absence of an effect of natural hormone variations during the natural menstrual cycle on the LINE-1 and Alu methylation levels in blood cells could be due to the relatively small samples size that was available for this study. Hereon, we could not exclude whether other non-studied repeats or single loci could be even more affected by other hormones (not only estrogen) in the cell lines studied or in blood cells. Future detailed genome wide studies are needed to explore this possibility.

In this study, we show that methylation at LINE-1 elements is independent of several factors. Indeed, we found that neither age nor natural hormone levels are modulating the methylation levels. However, gender remains an influential factor. These results highlight that variability of methylation at LINE-1 within one gender is stemming from three sources. First: experimental conditions related to the quantitative methods used. Second: environmental factors including diet, life styles and pollution. Third: genetic factors. How much the last two potential sources contribute to the variability of methylation at LINE-1 remains unknown. Our study eliminated potential factors that could affect the variability of LINE-1 methylation and it will pave the way for larger studies to examine, in more detail, the genetic and environmental effect on the levels of methylation of LINE-1 genome wide.

## Materials and Methods

### Ethics statement

All samples from all subjects were obtained upon written informed consent. The local Ethics Committee of the University Clinics of Bonn approved the study (approval numbers: 106/05 and 240/07).

### DNA samples

DNA from 500 healthy individuals distributed over all age groups (18–64 years old) was available for this study from blood donors attending the blood donation unit at the Institute of Experimental Hematology and Transfusion Medicine, University of Bonn. Additional samples from healthy female subjects were collected from young healthy volunteers (20–35 years old). Every female donor was asked to give blood three times per month for a duration of three to four months. The first blood sample was taken during the menstruation phase, the second near the end of the proliferative phase at the ovulation time (estimated from the cycle length) and the third at the middle of the secretory phase. Eleven, four and two women were able to give samples over four, three and two months, respectively. Only one woman withdrew from the study. From every blood donation, levels of estradiol, progesterone and testosterone were measured according to standard protocols.

### Methylation analysis

Bisulfite treatment was done using the EpiTect kit from Qiagen (Hilden, Germany) according to the manufacturer's procedure. PCRs were performed using previously published primers [25] and a hot-start Taq polymerase (HOT FIREPol from Solis BioDyne, Tartu, Estonia). Detailed information about the position of the primers used for the amplification of degenerate LINE-1 and Alu sequences can be found in Supplementary Figure S1. For accurate quantitative methylation analysis we used one of two assays, either a pyrosequencing based assay or the SIRPH protocol [47], [48], [49]. Quantitative methylation at one specific CpG site (CpG15 Supplementary Figure S1-A) at LINE-1 promoter was measured using the pyrosequencing technique (for age samples). This assay was chosen as it has a higher throughput and is easier to use for large number of samples.

Using the pyrosequencing assay we analyzed only one CpG site flanking the pyrosequencing primer. The reason for this is based on the fact that a pyrosequencing assay requires input of a DNA sequence to read the methylation levels while synthesizing the DNA strand according to the user-entered sequence. Since LINE-1 sequences are highly variable, we found that the longer the distance to the sequence start the greater the deviation from the consensus sequence and the less accurate the reflection on the actual real methylation levels. Supplementary Figure S5 that includes the sequencing of 50 clones illustrates this phenomenon. For only 50 sequenced clones, corresponding to different LINE-1 loci dispersed all over the genome, it would be possible to read the methylation of about 82%, 76%, 36% and 16% at CpGs 1, 2, 3 and 4, respectively. Already at CpG number 7 no clones could be read. Therefore and because of this bias, we studied only one CpG site directly flanking the pyrosequencing primer.

On the other hand, the SIRPH method was used for measuring methylation at two promoter specific LINE-1 CpG sites (SN-8 (CpG15) and SN-9; Supplementary Figure S1-A) and two Alu specific CpG sites (SN-1 and SN-4; Supplementary Figure S1-B) in the control samples of the menstrual cycle and the hormone-treated cell line [47], [48]. This assay was used for these samples because it is highly quantitative and very sensitive and can therefore detect very small variations of less than 1%. However, the throughput is less than with the pyrosequencing assay which makes SIRPH suitable for a relatively small number of samples.

### Cell culture and hormone treatment

#### Cell lines used in this study

Four different cell lines were used in this study: Hek293T (female), HUVEC (mixture of male and female), T47-Kbluc (female breast cancer cell line) [50] and MDA-kb2 (female breast cancer cell line) [51].

#### Culture conditions

Each cell line was cultivated in specific medium as follows: Hek293T were cultivated in DMEM Glc 1 g/l with L-Gln, T47D-KBluc were cultivated in RPMI 1640 with L-Glutamine, MDA-Kb2 cells were cultivated in Leibovitz L15, while HUVEC cells were cultivated in endothelial cell basal medium. All mediums were without phenol red and supplemented with 10% charcoal-treated FBS, penicillin 200 U/ml, streptomycin 0.5 mg/ml and amphotericin 25 µg/ml. The cells were treated with 4,5 alpha-Dihydrotestosterone (DHT; Sigma-Aldrich, ordering number: 10300) or with 17 beta-estradiol (E2; Sigma-Aldrich, ordering number: E2758) or with progesterone (4-Pregnenr-2,20-dione; Sigma-Aldrich, ordering number: P8783). While testosterone itself is a potent androgenic hormone, it nevertheless represents a prohormone that can be converted enzymatically into a threefold more potent androgen 5 alpha-DHT (5-alpha-reductase pathway) or even into estradiol (E2; aromatase or CYP19A1 pathway) [52]. Therefore, we used dihydrotestosterone instead of testosterone as an androgen due to its inability to enter the estrogen pathway of hormonal regulation. The different hormones were dissolved either in ethanol or in DMSO or in cyclodextrine ((2-Hydroxypropyl)- β- cyclodextrine; Sigma; product number C0926). A 30 µM working solution of each hormone was used to prepare a 1∶300 dilution in the culture medium for every specific cell line. This is corresponding to 0.1 nmole/ml of medium or to 27 ng/ml, 29 ng/ml and 31 ng/ml for estrogen, dihydrotestosterone and progesterone, respectively. In other words, this corresponds to about 68-fold, 36-fold and 1.8-fold the upper physiological concentration (in females) for estrogen, dihydrotestosterone and progesterone, respectively. For every assay, the cells were incubated in the respective hormones for 48 hours. A 48 hour treatment was chosen based on a previous report of a detectable effect of estrogen on chromatin modifications during this treatment time [53].

#### Luciferase assays

The responsiveness of the cell lines MDA-kb2 (responsive for testosterone) and T47D-KBluc (responsive for estradiol) for hormone treatment was measured by a luciferase assay as both cell lines contain a hormone responsive element upstream of luciferase reporter gene [50], [51]. The ‘Luciferase Reporter Gene Assay, high sensitivity’ from Roche was used (ordering number: 11 669 893 001). We treated the MDA-kb2 cells with either 100 nM DHT or with 100 nM E2 (only ethanol was used as negative control) and performed the luciferase assay. The response to DHT was about 14-fold higher than the ethanol blank, while the response to estrogen was about 4-fold higher than the blank. Induction of T47D-KBluc cells with E2 and DHT gave 7-fold and 2-fold higher response, respectively, than the ethanol blank control. We then tested different solvent mediums, namely ethanol, DMSO and cyclodextrine for the hormone preparation and found little differences between the three mediums (data not shown). Therefore, and for simplicity of preparation, we continued to use ethanol in preparation of the hormone working solutions.

#### RT-PCRs and western blots

RT-PCR and western blots where done according to standard procedures. Briefly, blood cells, HEK293, T47D, MDA and HUVEC cells were lysed in RIPA buffer (Sigma, Product No. R 0278) and the protein concentration was measured by Lowry assay. Equal amounts of each protein sample (30 ìg) were separated by electrophoresis on SDS-PAGE (BIORAD, Cat. No. 161-1101)) and blotted onto nitrocellulose membrane (GE Healthcare, Pack. No. RPN303F). Blots were incubated as indicated with primary antibodies raised against AR (Sigma, Product-No. A9853), and ER (recognizing both ERα and ERâ: Sigma, Product-No. E0521). The blots were developed with the chemiluminescent substrate solution CDP-*Star* ^TM^ (Sigma, product No. C0712) using specific alkaline phosphatase-conjugated anti-IgG specific antibodies. Primers used for the RT-PCRs are listed in Supplementary Table S1.

### Statistical analysis

#### Correction of variation between different plates and trend line within a given plate

Since the number of samples used to study the effect of age was too large for analysis in one 96-well plate, the samples had to be analyzed over several plates. This resulted in differences in the general average between plates, which had to be corrected before all samples were pooled together for further statistical evaluations. The two observed deviations were a trend line of decrease or increase over a plate (due to processing time) and different average values between plates. These observed experimental issues were corrected as follows: A linear regression for the trend line within each plate was calculated and the measured methylation values were corrected accordingly, so that after correction the linear regression of a given plate was parallel to the X-axis. In a final step, the individual plates were normalized according to the global average. This correction of experimentally introduced biases did not change the expected results of the controls in the factor VIII region. Therefore, we expect that this correction will have a minor effect (if any) on other tested regions. The data before and after correction are represented by box plots in Supplementary Figure S6-A, the age distribution in every experimental group is shown in Supplementary Figure S6-B.

#### Correlation with age and gender

Pearson correlation was applied to test the correlation between age and methylation, while the gender effect was tested by t-test.

#### Effect of methylation and hormones on each other and during different stages of the menstrual cycle

In this study, we repeatedly (over a 3-4 months period) measured hormone and methylation levels at selected CpG sites in different female donors at three different time points during the menstrual cycle. The dependence of repeated measurements in each particular female blood donor violates a basic assumption in the linear regression model, which ignores dependent observations on the same subject. On the other hand, by using an ANOVA for repeated measurements or a linear regression model, an estimation of the variability for all samples could be given. However, a measure of between-subject variability is not integrated. Since measurements in one woman are expected to be much more similar than measurements in different women, regression and ANOVA require an extension that incorporates the within-subject dependence. Therefore, a mixed linear model seemed to be the most appropriate due to the structure of the data. The co-variance structure is specified by standard variance components. In this model, the women and their sampling dates (during the month) represent the additional random effects and the fixed effect is either the methylation or the hormone level.

#### Variability of the methylation between different female donors

To examine differences (if any) in methylation at L1 (at two CpG sites) and Alu (at two CpG sites) between different women we used the ANOVA model. For this calculation, we considered the methylation value as dependent variable and the women as effect.

If inter-individual differences between different women exist the variability of methylation between women should be higher than the variability of different measurements in one woman. Therefore, by calculating the variability between different women and within each woman we could deduce the stability of inter-individual methylation differences.

## Supporting Information

Figure S1 Bisulfite converted and normal sequence of studied Line-1 (A), Alu (B) repeats and F8 (C), the used PCR, SIRPH or pyrosequencing primers sequences are labeled.
(PDF)

Figure S2 In this diagram, the effect of annealing temperature on average methylation as measured by SIRPH reaction is shown. The higher the annealing temperature used the bigger is the difference between males and females for this CpG site in LINE-1 sequence. Four males and four females aged 21 years were used for this experiment.
(PDF)

Figure S3 50 sequenced clone of LINE-1 derived from male peripheral blood derived PCR products (Line-1 degenerate primers). The consensus sequence is shown in bold below the sequences. This figure shows the polymorphisms at CpG sites, the non CpG/TpG dinucleotide at the CpGs number 15, 16, 17, 18, 19, 20, 21 and 22 are highlighted in the red boxes. The oval boxes correspond to deviations from the consensus sequence at non CpG sites. Deviations from the consensus sequence that is used by the pyrosequencer to read the methylation averages causes the synthesis of the DNA strands to hold or to incorporate the wrong nucleotide in the wrong timing thus affecting the quantitative reading of the methylation values. This is particularly illustrated by the percentage of readable CpGs at every site which start with 82% at position 1 (CpG15) and end with 0% at position 7 (CpG21).
(PDF)

Figure S4 A) Methylation value distribution in the six experimental groups before and after correction. In every group, the number of males and females is also shown. B) Age distribution in the six experimental groups. In every group the number of males and females is also shown.
(PDF)

Table S1 Primer sequences used in this study.
(PDF)

Table S2 Summary findings of several studies that analyzed LINE-1 global methylation in healthy human individuals.
(PDF)
