# Correction: Methylation at Global LINE-1 Repeats in Human Blood Are Affected by Gender but Not by Age or Natural Hormone Cycles

**DOI:** 10.1371/journal.pone.0119057

**Published:** 2015-03-05

**Authors:** The PLOS ONE Staff

There is an error in the fourth sentence of the second paragraph under the “Methylation analysis” section of Materials and Methods. The correct sentence is: Supplementary Figure S3 that includes the sequencing of 50 clones illustrates this phenomenon.
