# Supplementary material for: Methylation at Global LINE-1 Repeats in Human Blood Are Affected by Gender but Not by Age or Natural Hormone Cycles
Source: PLoS One. 2011 Jan 19;6(1):e16252. doi: 10.1371/journal.pone.0016252 (PMC3023801; doi:10.1371/journal.pone.0016252)
Supplement: Figure S1 — Bisulfite converted and normal sequence of studied Line-1 (A), Alu (B) repeats and F8 (C), the used PCR, SIRPH or pyrosequencing primers sequences are labeled. (PDF) [file pone.0016252.s001.pdf]

A

Line-1 global

Line 1 elements (accession number X52235) Wild type sequence

```
 61 CGcagaagac gggtgatttc tgcatttcca tctgaggtac tgggttcatc tcactaggga
                     1                2 3   4       6   7        8
121 gtgccagaca gtgggCGcag gtcagtgggt gCGCGcacCG tgcaCGagcC GaagcagggC
                           10                      11
181 Gaggcattgt ctcacttggg aagCGcaagg ggtcagggag ttccctttcC Gagtcaaaga
              12   13                14               15       16    17
241 aaggggtgaC GgaCGcacct ggaaaatCGg gtctctccca ccCGaatatt gCGctttCGg
      18              19 20   21
301 acCGgcttaa aaaaCGgCGc acCGCGagat tatatcttgc acctggctag gagggtccta
    24    25      26                                              27
361 CGcccaCGga gtctCGctga ttgctagcac agcagtctga gatcaaactg caaggcCGca
                     29   30
421 gcaaggctgg gggaggggCG ccCGccattg cccaggcttg cttgggtaaa caaagcagcc
            32
481 tggcagctCG aactgggtgg agcccaccac agctcaagga ggcctgcctg cctttgtagg
```

Line 1 elements (accession number X52235) Bisulfite sequence

```
                                                     Bi-L1-F1
 61 CGtagaagat gggtgatttt tgtattttta tttgaggtat tgggtttatt ttattaggga SN-9
                     1                2 3   4       6   7        8
121 gtgttagata gtgggCGtag gttagtgggt gCGCGtatCG tgtaCGagtC GaagtagggC
                           10                      11
181 Gaggtattgt tttatttggg aagCGtaagg ggttagggag tttttttttC Gagttaaaga
              12   13                14      SN-8     15       16    17
241 aaggggtgaC GgaCGtattt ggaaaatCGg gtttttttta ttCGaatatt gCGttttCGg
      18              19 20   21
301 atCGgtttaa aaaaCGgCGt atCGCGagat tatattttgt atttggttag gagggtttta
    24    25      26                                              27
361 CGtttaCGga gtttCGttga ttgttagtat agtagtttga gattaaattg taaggtCGta
                     29   30
421 gtaaggttgg gggaggggCG ttCGttattg tttaggtttg tttgggtaaa taaagtagtt
            32
481 tggtagttCG aattgggtgg agtttattat agtttaagga ggtttgtttg tttttgtagg
                   Bi-L1-R2
```

Pyrosequencing primer: SN-8; SIRPH Primer: SN-8 and SN-9

Supplementary Figure S1-A: LINE-1 global specific primers. CpG numbering is not continuous because of gaps in the consensus sequence when compared to the sequenced LINE-1 clones.

B

Alu Global

Alu (accession numbers: Alu-J U14567; Alu-Sb U14568; Alu-Sb1 U14569; Alu-Sb2 U14570; Alu-Sc U14571; Alu-Sp U14572; Alu-Sq U14573; Alu-Sx U14574)

<u>Wild type sequences alignment</u>

```
                                                                          1
Sp-Alu          ----------GGCCGGGCGCGGTGGCTCACGCCTGTAATCCCAGCACTTTGGGAGGCCGA 50
Sq-Alu          ----------GGCCGGGCGCGGTGGCTCACGCCTGTAATCCCAGCACTTTGGGAGGCCGA 50
Sx-Alu          GGCGGGCGGAGGCCGGGCGCGGTGGCTCACGCCTGTAATCCCAGCACTTTGGGA------ 54
J-Alu           ----------GGCCGGGCGCGGTGGCTCACGCCTGTAATCCCAGCACTTTGGGAGGCCGA 50
Sb1-Alu         ----------GGCCGGGCGCGGTGGCTCACGCCTGTAATCCCAGCACTTTGGGAGGCCGA 50
Sb2-Alu         ----------GGCCGGGCGCGGTGGCTCACGCCTGTAATCCCAGCACTTTGGGAGGCCGA 50
Sb-Alu          ----------GGCCGGGCGCGGTGGCTCACGCCTGTAATCCCAGCACTTTGGGAGGCCGA 50
Sc-Alu          ----------GGCCGGGCGCGGTGGCTCACGCCTGTAATCCCAGCACTTTGGGAGGCCGA 50
                          ********************************************

                  2   3         4    5        6          7          8
Sp-Alu          GGCGGGCGGATCACCTGAGGTCGGGAGTTCGAGACCAGCCTGACCAACATGGAGAAACCC 110
Sq-Alu          GGCGGGTGGATCACCTGAGGTCAGGAGTTCGAGACCAGCCTGGCCAACATGGTGAAACCC 110
Sx-Alu          ----GGAAGATCACCTGAGGTCAGGAGTTCGAGACCAGCCTGGCCAACATGGTGAAACCC 110
J-Alu           GGCGGGAGGATCACTTGAGCCCAGGAGTTCGAGACCAGCCTGGGCAACATAGTGAAACCC 110
Sb1-Alu         GGCGGGCGGATCA--CGAGGTCAGGAGATCGAGACCATCCCGGCTAAAACGGTGAAACCC 108
Sb2-Alu         GGCGGGTGGATCA--TGAGGTCAGGAGATCGAGACCATCCTGGCTAACAAGGTGAAACCC 108
Sb-Alu          GGCGGGCGGATCA--CGAGGTCAGGAGATCGAGACCATCCTGGCTAACACGGTGAAACCC 108
Sc-Alu          GGCGGGCGGATCA--CGAGGTCAAGAGATCGAGACCATCCTGGCCAACATGGTGAAACCC 108
                    **  *****   ***  *  *** ********* ** *   ** *  * *******

                9                            10  11 12    131415
Sp-Alu          CGTCTCTACTAAAAATACAAAAA-TTAGCCGGGCGTGGTGGCGCATGCCTGTAATCCCAG 169
Sq-Alu          CGTCTCTACTAAAAATACAAAAA-TTAGCCGGGCGTGGTGGCGGGCGCCTGTAATCCCAG 169
Sx-Alu          CGTCTCTACTAAAAATACAAAAA-TTAGCCGGGCGTGGTGGCGCGCGCCTGTAATCCCAG 169
J-Alu           CGTCTCTACAAAAAATACAAAAA-TTAGCCGGGCGTGGTGGCGCGCGCCTGTAGTCCCAG 169
Sb1-Alu         CGTCTCTACTAAAAATACAAAAA-TTAGCCGGGCGTAGTGGCGGGCGCCTGTAGTCCCAG 167
Sb2-Alu         CGTCTCTACTAAAAATACAAAAAATTAGCCGGGCGCGGTGGCGGGCGCCTGTAGTCCCAG 168
Sb-Alu          CGTCTCTACTAAAAATACAAAAA-TTAGCCGGGCGTGGTGGCGGGCGCCTGTAGTCCCAG 167
Sc-Alu          CGTCTCTACTAAAAATACAAAAA-TTAGCTGGGCGTGGTGGCGCGCGCCTGTAGTCCCAG 167
                ********* ************* ***** *****  ******   ******* ******

                     16                    17 18     19      20         21
Sp-Alu          CTACTCGGGAGGCTGAGGCAGGAGAATCGCTTGAACCCGGGAGGCGGAGGTTGCGGTGAG 229
Sq-Alu          CTACTCGGGAGGCTGAGGCAGGAGAATCGCTTGAACCCGGGAGGCGGAGGTTGCAGTGAG 229
Sx-Alu          CTACTCGGGAGGCTGAGGCAGGAGAATCGCTTGAACCCGGGAGGCGGAGGTTGCAGTGAG 229
J-Alu           CTACTCGGGAGGCTGAGGCAGGAGGATCGCTTGAGCCCGGGAGGTCGAGGCTGCAGTGAG 229
Sb1-Alu         CTACTTGGGAGGCTGAGGCAGGAGAATGGCGTGAACCCGGGAGGCGGAGCTTGCAGTGAG 227
Sb2-Alu         CTACTGGGGAGGCTGAGGCAGGAGAATGGCGTGAACCCGGGAAGCGGAGCTTGCAGTGAG 228
Sb-Alu          CTACTCGGGAGGCTGAGGCAGGAGAATGGCGTGAACCCGGGAGGCGGAGCTTGCAGTGAG 227
Sc-Alu          CTACTCGGGAGGCTGAGGCAGGAGAATCGCTTGAACCCGGGAGGCGGAGGTTGCAGTGAG 227
                ***** *****************  ** ** *** ******* *  ***  *** *****

                 22   23 24
Sp-Alu          CCGAGATCGCGCCATTGCACTCCA-------GCCTGGGCAACAAGAGCGAAACTCCGTCT 282
Sq-Alu          CCGAGATCGCGCCACTGCACTCCA-------GCCTGGGCAACAAGAGCGAAACTCCGTCT 282
Sx-Alu          CCGAGATCGCGCCACTGCACTCCA-------GCCTGGGCGACA-GAGCGAGACTCCGTCT 281
J-Alu           CCGTGATCGCGCCACTGCACTCCA-------GCCTGGGCGACA-GAGCGAGACCCTGTCT 281
Sb1-Alu         CCGAGATCCCGCCACTGCACTCCA-------GCCTGGGCGACA-GAGCGAGACTCCGTCT 279
Sb2-Alu         CCGAGATTGCGCCACTGCAGTCCGCAGTCCGGCCTGGGCGACA-GAGCGAGACTCCGTCT 287
Sb-Alu          CCGAGATCGCGCCACTGCACTCCA-------GCCTGGGCGACA-GAGCGAGACTCCGTCT 279
Sc-Alu          CCGAGATCGCGCCACTGCACTCCA-------GCCTGG-CGACA-GAGCGAGACTCCGTCT 278
                *** ***  ***** **** ***        ****** * *** ****** ** * ****

Sp-Alu          CAAAAAAAA 291
Sq-Alu          CAAAAAAAA 291
Sx-Alu          CAAAAAAAA 290
J-Alu           CAAAAAAAA 290
Sb1-Alu         CAAAAAAAA 288
Sb2-Alu         CAAAAAAAA 296
Sb-Alu          CAAAAAAAA 288
Sc-Alu          CAAAAAAAA 287
                *********
```

Supplementary Figure S1-B: Alu global specific primers (continued on next page)

Bisulfite sequences alignment

```
                                                  Alu-AII-F3         1
Sp-Alu          ----------GGTCGGGCGTGGTGGTTTACGTTTGTAATTTTAGTATTTTGGGAGGTCGA 50
Sq-Alu          ----------GGTCGGGCGTGGTGGTTTACGTTTGTAATTTTAGTATTTTGGGAGGTCGA 50
Sx-Alu          GGCGGGCGGAGGTCGGGCGTGGTGGTTTACGTTTGTAATTTTAGTATTTTGGGA------ 54
J-Alu           ----------GGTCGGGCGTGGTGGTTTACGTTTGTAATTTTAGTATTTTGGGAGGTCGA 50
Sb1-Alu         ----------GGTCGGGCGTGGTGGTTTACGTTTGTAATTTTAGTATTTTGGGAGGTCGA 50
Sb2-Alu         ----------GGTCGGGCGTGGTGGTTTACGTTTGTAATTTTAGTATTTTGGGAGGTCGA 50
Sb-Alu          ----------GGTCGGGCGTGGTGGTTTACGTTTGTAATTTTAGTATTTTGGGAGGTCGA 50
Sc-Alu          ----------GGTCGGGCGTGGTGGTTTACGTTTGTAATTTTAGTATTTTGGGAGGTCGA 50
                          ********************************************

                  2    3        4     5       6          7        8
Sp-Alu          GGCGGGCGGATTATTTGAGGTCGGGAGTTCGAGATTAGTTTGATTAATATGGAGAAATTT 110
Sq-Alu          GGCGGGTGGATTATTTGAGGTTAGGAGTTCGAGATTAGTTTGGTTAATATGGTGAAATTT 110
Sx-Alu          ----GGAAGATTATTTGAGGTTAGGAGTTCGAGATTAGTTTGGTTAATATGGTGAAATTT 110
J-Alu           GGCGGGAGGATTATTTGAGTTTAGGAGTTCGAGATTAGTTTGGGTAATATAGTGAAATTT 110
Sb1-Alu         GGCGGGCGGATTA--CGAGGTTAGGAGATCGAGATTATTTCGGTTAAAACGGTGAAATTT 108
Sb2-Alu         GGCGGGTGGATTA--TGAGGTTAGGAGATCGAGATTATTTTGGTTAATAAGGTGAAATTT 108
Sb-Alu          GGCGGGCGGATTA--CGAGGTTAGGAGATCGAGATTATTTTGGTTAATACGGTGAAATTT 108
Sc-Alu          GGCGGGCGGATTA--CGAGGTTAAGAGATCGAGATTATTTTGGTTAATATGGTGAAATTT 108
                    **  *****   *** *   *** * ******* ** *  *** *  * *******

                9             SN-4            10  11 12    131415
Sp-Alu          CGTTTTTATTAAAAATATAAAAA-TTAGTCGGGCGTGGTGGCGTATGTTTGTAATTTTAG 169
Sq-Alu          CGTTTTTATTAAAAATATAAAAA-TTAGTCGGGCGTGGTGGCGGGCGTTTGTAATTTTAG 169
Sx-Alu          CGTTTTTATTAAAAATATAAAAA-TTAGTCGGGCGTGGTGGCGCGCGTTTGTAATTTTAG 169
J-Alu           CGTTTTTATAAAAAATATAAAAA-TTAGTCGGGCGTGGTGGCGCGCGTTTGTAGTTTTAG 169
Sb1-Alu         CGTTTTTATTAAAAATATAAAAA-TTAGTCGGGCGTAGTGGCGGGCGTTTGTAGTTTTAG 167
Sb2-Alu         CGTTTTTATTAAAAATATAAAAAATTAGTCGGGCGCGGTGGTGGGCGTTTGTAGTTTTAG 168
Sb-Alu          CGTTTTTATTAAAAATATAAAAA-TTAGTCGGGCGTGGTGGCGGGCGTTTGTAGTTTTAG 167
Sc-Alu          CGTTTTTATTAAAAATATAAAAA-TTAGTTGGGCGTGGTGGCGCGCGTTTGTAGTTTTAG 167
                ********* ************* ***** *****  **** *   ******* ******

                     16          SN-1         17 18     19      20       21
Sp-Alu          TTATTCGGGAGGTTGAGGTAGGAGAATCGTTTGAATTCGGGAGGCGGAGGTTGCGGTGAG 229
Sq-Alu          TTATTCGGGAGGTTGAGGTAGGAGAATCGTTTGAATTCGGGAGGCGGAGGTTGTAGTGAG 229
Sx-Alu          TTATTCGGGAGGTTGAGGTAGGAGAATCGTTTGAATTCGGGAGGCGGAGGTTGTAGTGAG 229
J-Alu           TTATTCGGGAGGTTGAGGTAGGAGGATCGTTTGAGTTCGGGAGGTCGAGGTTGTAGTGAG 229
Sb1-Alu         TTATTTGGGAGGTTGAGGTAGGAGAATGGCGTGAATTCGGGAGGCGGAGTTTGTAGTGAG 227
Sb2-Alu         TTATTGGGGAGGTTGAGGTAGGAGAATGGCGTGAATTCGGGAAGCGGAGTTTGTAGTGAG 228
Sb-Alu          TTATTCGGGAGGTTGAGGTAGGAGAATGGCGTGAATTCGGGAGGCGGAGTTTGTAGTGAG 227
Sc-Alu          TTATTCGGGAGGTTGAGGTAGGAGAATCGTTTGAATTCGGGAGGCGGAGGTTGTAGTGAG 227
                ***** ****************** ** *  *** ******* *  *** ***  *****

                 22   23 24         Alu-AII-R2
Sp-Alu          TCGAGATCGCGTTATTGTATTTTA-------GTTTGGGTAATAAGAGTGAAATTTCGTTT 282
Sq-Alu          TCGAGATCGCGTTATTGTATTTTA-------GTTTGGGTAATAAGAGTGAAATTTCGTTT 282
Sx-Alu          TCGAGATCGCGTTATTGTATTTTA-------GTTTGGGCGATA-GAGCGAGATTTCGTTT 281
J-Alu           TCGTGATCGCGTTATTGTATTTTA-------GTTTGGGCGATA-GAGCGAGATTTTGTTT 281
Sb1-Alu         TCGAGATTTCGTTATTGTATTTTA-------GTTTGGGCGATA-GAGCGAGATTTCGTTT 279
Sb2-Alu         TTGAGATTGCGTTATTGTAGTTCGTAGTTCGGTTTGGGCGATA-GAGCGAGATTTCGTTT 287
Sb-Alu          TCGAGATCGCGTTATTGTATTTTA-------GTTTGGGCGATA-GAGCGAGATTTCGTTT 279
Sc-Alu          TCGAGATCGCGTTATTGTATTTTA-------GTTTGG-CGATA-GAGCGAGATTTCGTTT 278
                * * ***  ********** **         ******   *** *** ** **** ****

Sp-Alu          TAAAAAAAA 291
Sq-Alu          TAAAAAAAA 291
Sx-Alu          TAAAAAAAA 290
J-Alu           TAAAAAAAA 290
Sb1-Alu         TAAAAAAAA 288
Sb2-Alu         TAAAAAAAA 296
Sb-Alu          TAAAAAAAA 288
Sc-Alu          TAAAAAAAA 287
                *********
```

SIRPH primer: SN-1 and SN-4

Supplementary Figure S1-B: Alu global specific primers (continued from previous page)

C

Factor 8 region Xq28

Factor 8 (accession number: M88640) Wild type sequence

3061 agttcacatt tatcagaagg acctattccc taCGgaaact agcaatgggt ctcctggcca
1
3121 tctggatctC Gtggaaggga gccttcttca gggaacagag ggagCGatta agtggaatga
3181 agcaaacaga cctggaaaag ttccctttct gagagtagca acagaaagct ctgcaaagac
3241 tccctccaag ctattggatc ctcttgcttg ggataaccac tatggtactc agataccaaa
3301 agaagagtgg aaatcccaag agaagtcacc agaaaaaaca gcttttaaga aaaaggatac
2
3361 cattttgtcc ctgaaCGctt gtgaaagcaa tcatgcaata gcagcaataa atgagggaca
3
3421 aaataagccC Gaaatagaag tcacctgggc aaagcaaggt aggactgaaa ggctgtgctc
4 5 6
3481 tcaaaaccca ccagtcttga aaCGccatca aCGggaaata actCGtacta ctcttcagtc
3541 agatcaagag gaaattgact atgatgatac catatcagtt gaaatgaaga aggaagattt
7 8
3601 tgacatttat gatgaggatg aaaatcagag ccccCGcagc tttcaaaaga aaacaCGaca
3661 ctattttatt gctgcagtgg agaggctctg ggattatggg atgagtagct ccccacatgt

Factor 8 (accession number: M88640) Bisulfite treated sequence

3061 agtttatatt tattagaagg atttattttt taCGgaaatt agtaatgggt tttttggtta
BiF8-F6 1 **BiF8-F5**
3121 tttggattC Gtggaaggga gtttttttta gggaatagag ggagCGatta agtggaatga
3181 agtaaataga tttggaaaag tttttttttt gagagtagta atagaaagtt ttgtaaagat
3241 ttttttttaag ttattggatt tttttgtttg ggataattat tatggtattt agatattaaa
3301 agaagagtgg aaattttaag agaagttatt agaaaaaata gtttttaaga aaaaggatat
2
3361 tattttgttt ttgaaCGttt gtgaaagtaa ttatgtaata gtagtaataa atgagggata
3
3421 aaataagttC Gaaatagaag ttatttgggt aaagtaaggt aggattgaaa ggttgtgttt
4 5 6
3481 ttaaaattta ttagttttga aaCGttatta aCGggaaata attCGtatta ttttttagtt
3541 agattaagag gaaattgatt atgatgatat tatattagtt gaaatgaaga aggaagattt
SN-1 7 8
3601 tgatatttat gatgaggatg aaaattagag ttttCGtagt ttttaaaaga aaataCGata
3661 ttattttatt gttgtagtgg agaggttttg ggattatggg atgagtagtt ttttatatgt
BiF8-R5 **BiF8-R6**

Pyrosequencing primer: SN-1

Supplementary Figure S1-C: F8 region specific primers
