# Supplementary material for: Methylation at Global LINE-1 Repeats in Human Blood Are Affected by Gender but Not by Age or Natural Hormone Cycles
Source: PLoS One. 2011 Jan 19;6(1):e16252. doi: 10.1371/journal.pone.0016252 (PMC3023801; doi:10.1371/journal.pone.0016252)
Supplement: Figure S2 — In this diagram, the effect of annealing temperature on average methylation as measured by SIRPH reaction is shown. The higher the annealing temperature used the bigger is the difference between males and females for this CpG site in LINE-1 sequence. Four males and four females aged 21 years were used for this experiment. (PDF) [file pone.0016252.s002.pdf]

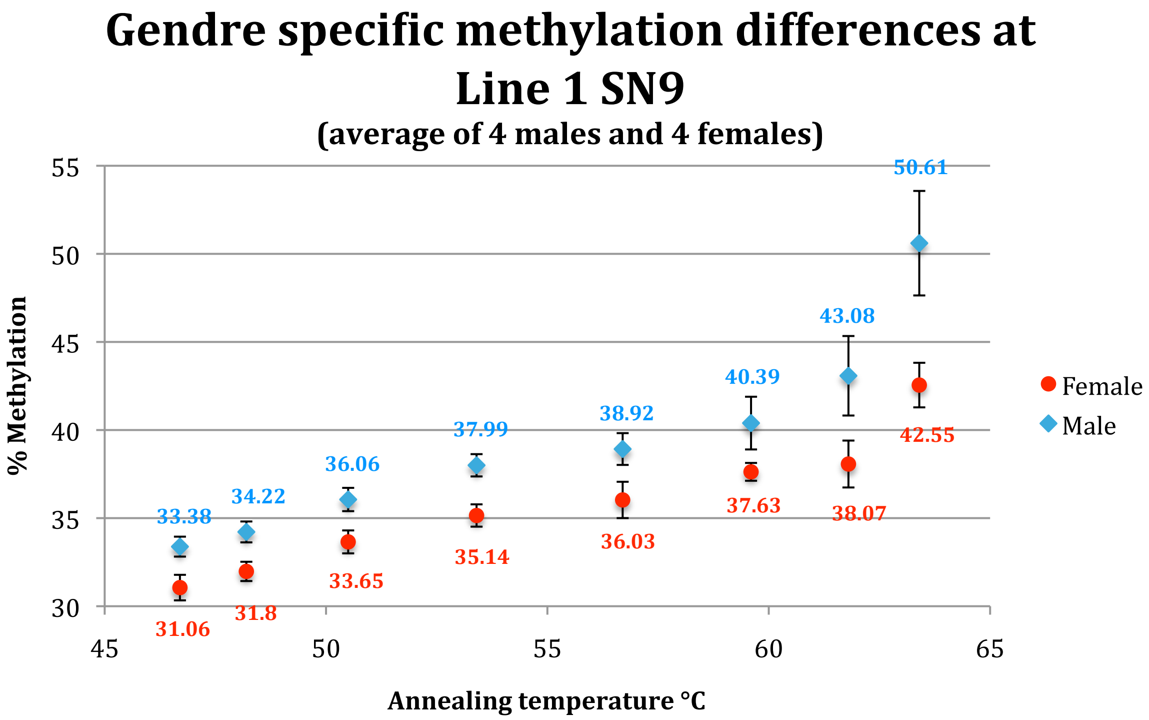

Supplementary Figure S2: In this diagram, the effect of annealing temperature on average methylation as measured by SIRPH reaction is shown. The higher the annealing temperature used the bigger the difference between males and females for this CpG site in LINE-1 sequence. Four males and four females aged 21 years were used for this experiment.
