# Supplementary material for: Methylation at Global LINE-1 Repeats in Human Blood Are Affected by Gender but Not by Age or Natural Hormone Cycles
Source: PLoS One. 2011 Jan 19;6(1):e16252. doi: 10.1371/journal.pone.0016252 (PMC3023801; doi:10.1371/journal.pone.0016252)
Supplement: Figure S3 — 50 sequenced clone of LINE-1 derived from male peripheral blood derived PCR products (Line-1 degenerate primers). The consensus sequence is shown in bold below the sequences. This figure shows the polymorphisms at CpG sites, the non CpG/TpG dinucleotide at the CpGs number 15, 16, 17, 18, 19, 20, 21 and 22 are highlighted in the red boxes. The oval boxes correspond to deviations from the consensus sequence at non CpG sites. Deviations from the consensus sequence that is used by the pyrosequencer to read the methylation averages causes the synthesis of the DNA strands to hold or to incorporate the wrong nucleotide in the wrong timing thus affecting the quantitative reading of the methylation values. This is particularly illustrated by the percentage of readable CpGs at every site which start with 82% at position 1 (CpG15) and end with 0% at position 7 (CpG21). (PDF) [file pone.0016252.s003.pdf]

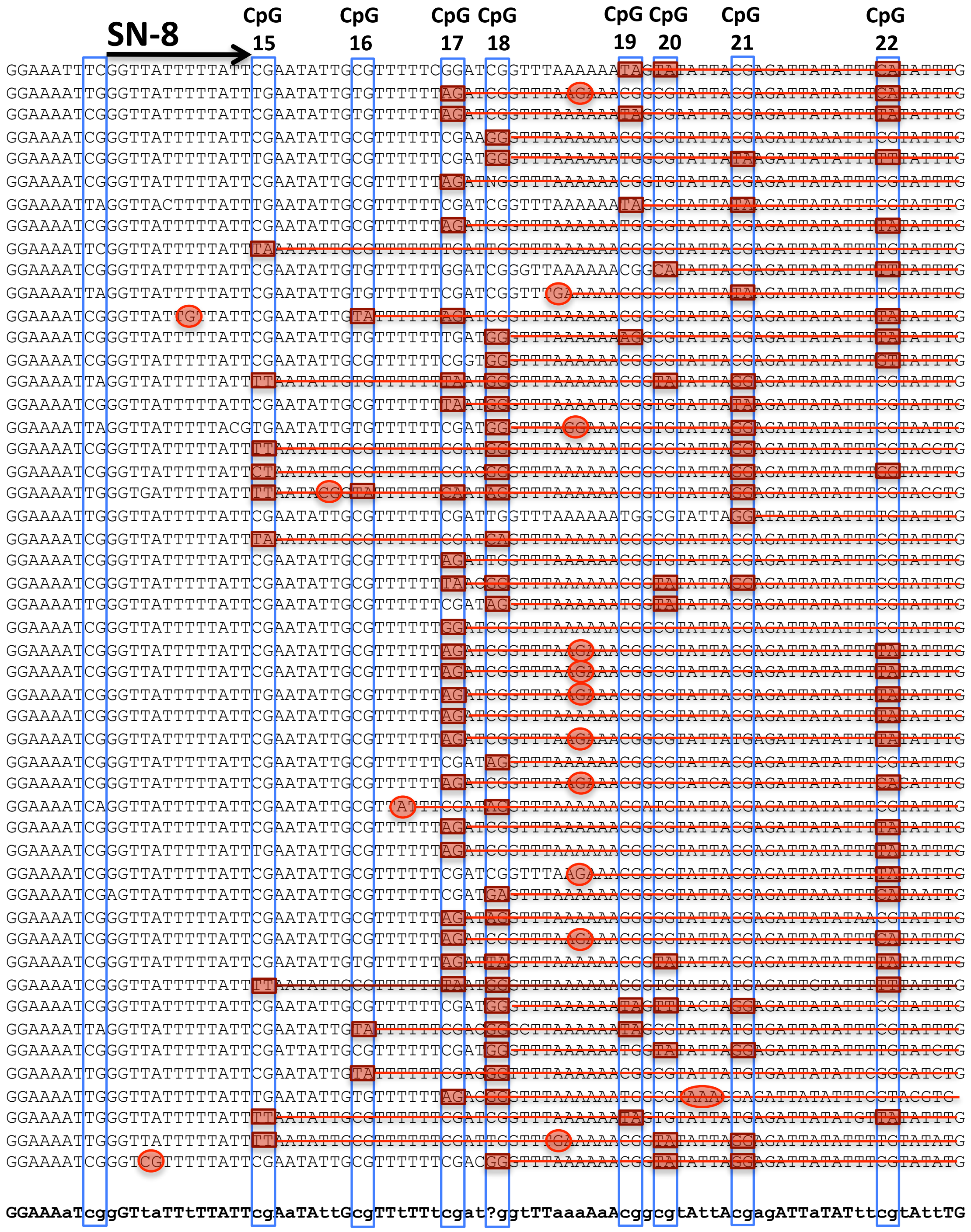

Readable clones	CpG 15	CpG 16	CpG 17	CpG 18	CpG 19	CpG 20	CpG 21	CpG 22
	41	38	18	8	2	1	0	0 / 50
	82%	76%	36%	16%	4%	2%	0%	0%

Supplementary Figure S3: 50 sequenced clone of LINE-1 derived from male peripheral blood derived PCR products (Line-1 degenerate primers). The consensus sequence is shown in bold below the sequences. This figure shows the polymorphisms at CpG sites, the non CpG/TpG dinucleotide at the CpGs number 15, 16, 17, 18, 19, 20, 21 and 22 are highlighted in the red boxes. The oval boxes correspond to deviations from the consensus sequence at non CpG sites. Horizontal lines indicate the unreadable sequence after a given polymorphism. Deviations from the consensus sequence that is used by the pyrosequencer to read the methylation averages causes the synthesis of the DNA strands to hold or to incorporate the wrong nucleotide in the wrong timing thus affecting the quantitative reading of the methylation values. This is particularly illustrated by the percentage of readable CpGs at every site which start with 82% at position 1 (CpG15) and end with 0% at position 7 (CpG21).
