# Supplementary material for: Methylation at Global LINE-1 Repeats in Human Blood Are Affected by Gender but Not by Age or Natural Hormone Cycles
Source: PLoS One. 2011 Jan 19;6(1):e16252. doi: 10.1371/journal.pone.0016252 (PMC3023801; doi:10.1371/journal.pone.0016252)
Supplement: Figure S4 — A) Methylation value distribution in the six experimental groups before and after correction. In every group, the number of males and females is also shown. B) Age distribution in the six experimental groups. In every group the number of males and females is also shown. (PDF) [file pone.0016252.s004.pdf]

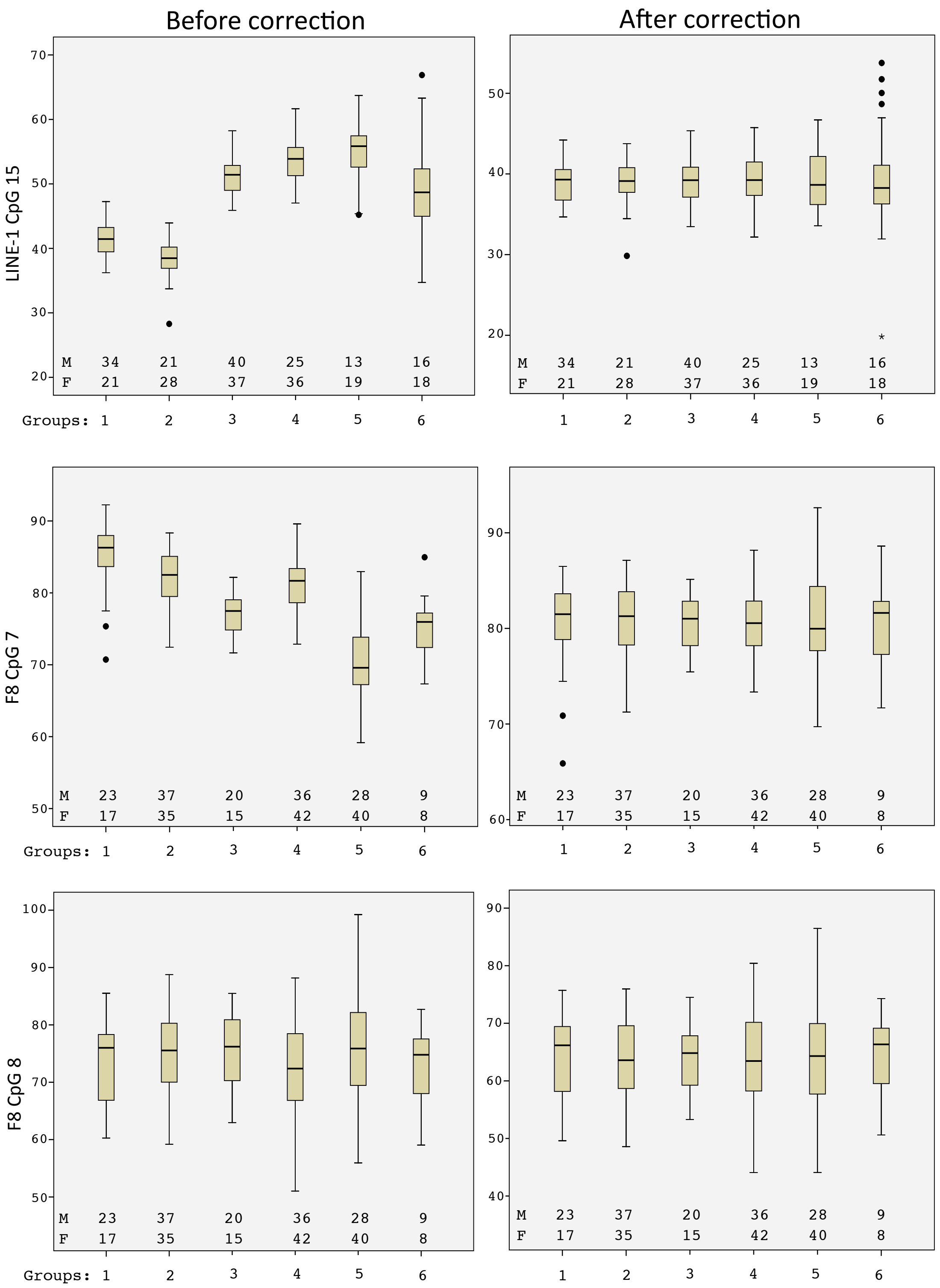

Supplementary Figure S4: A) Methylation values distribution in the six experimental groups before and after the correction. In every group, the number of males and females is also shown.

LINE-1 samples age distribution

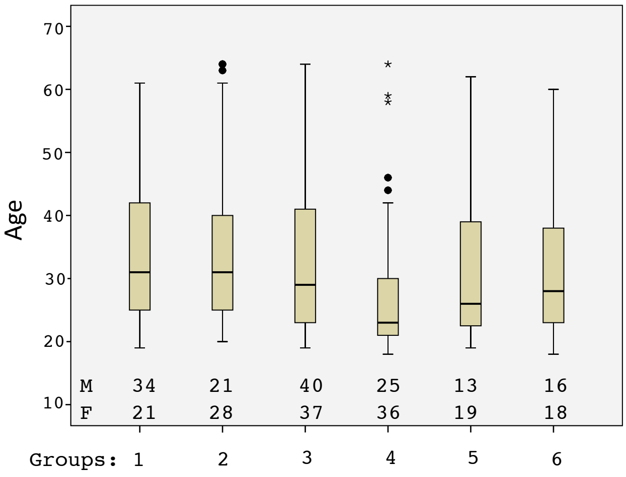

F8 samples age distribution

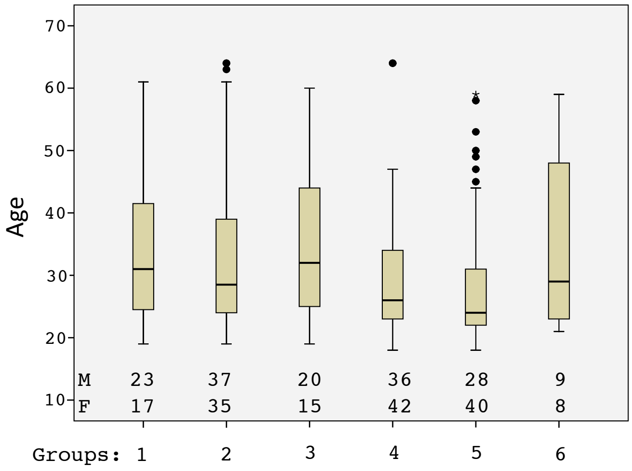

Supplementary figure S4: B) Age distribution in the six experimental groups. In every group, the number of males and females is also shown.
