# Supplementary material for: Methylation at Global LINE-1 Repeats in Human Blood Are Affected by Gender but Not by Age or Natural Hormone Cycles
Source: PLoS One. 2011 Jan 19;6(1):e16252. doi: 10.1371/journal.pone.0016252 (PMC3023801; doi:10.1371/journal.pone.0016252)
Supplement: Table S1 — Primer sequences used in this study. (PDF) [file pone.0016252.s005.pdf]

Supplementary Table S1: primer sequences used in this study

bisulfite primer				Pyrosequencing/SIRPH	
**Name**	**Sequence 5´to 3**	**Annealing**	**Product**	**Name**	**Sequence**
**Bisulfite**					
L1 repeats, Genome wide, X58075 (Suppl Fig S1)					
Bi-L1-F1	att tta tta ggg agt gtt aga tag tg	59°C	410 bp	SN-L1-8 (Pyro)	ttt ggg ttt ttt tta tt
Bi-L1-R2	tta aac tat aat aaa ctc cac cca at			SN-L1-9	ggg agt gtt aga tag tgg g
Alu repeats, Genome wide, Consensus (Suppl Fig S1)					
Bi-Alu-All-F3	ttg taa ttt tag tat ttt ggg	49°C	237 bp	SN-Alu-1	(ttt t)ag gtt gag gta gga gaa t
Bi-Alu-All-R2	cca aac taa aat aca ata a			SN-Alu-4	(ttt ttt) ttt tta tta aaa ata taa aaa tta gt
F8-ex14, Xq28, M88640					
Bi-F8Ex14-F5	tag taa tgg gtt ttt tgg tta ttt gga t	50°C	617 bp	SN-F8-1	gat gaa aat tag agt ttt
Bi-F8Ex14-R6	taa aaa act act cat ccc ata atc cca a				
Bi-F8Ex14-F6	agg gag ttt ttt tta ggg aat aga ggg a	55°C	561 bp		
Bi-F8Ex14-R5	taa tcc caa aac ctc tcc act aca aca a				
**RT-PCRs**					
Androgen receptor, Xq11.2-q12, NM_000044.2					
R-AR-F1	atg tcc tgg aag cca ttg ag	61°C	323 bp		
R-AR-R1	gct gta cat ccg gga ctt gt				
Estrogen receptor 1, 6q25.1, NM_000125.3					
R-ERalpha-F1	aca agc gcc aga gag atg at	61°C	361 bp		
R-ERalpha-R1	agg atc tct agc cag gca ca				
Estrogen receptor 2, 14q23.2, NM_001040275.1					
R-ERbeta-F	caa tcc atc tta ccc ctg ga	59°C	379 bp		
R-ERbeta-R	cat ccc tct tgg aac ctg ga				
Progestron receptor, 11q22-23, NM_000926.4					
R-PR-F1	tgg aag aaa tga ctg cat cg	59°C	363 bp		
R-PR-R1	tgc ctc tcg cct agt tga tt				
GAPDH control					
RT-GAPDH-F	tca cca ggg ctg ctt tta ac	59°C	508 bp		
RT-GAPDH-R	gtc ttc tgg gtg gca gtg at				
