# Supplementary material for: Methylation at Global LINE-1 Repeats in Human Blood Are Affected by Gender but Not by Age or Natural Hormone Cycles
Source: PLoS One. 2011 Jan 19;6(1):e16252. doi: 10.1371/journal.pone.0016252 (PMC3023801; doi:10.1371/journal.pone.0016252)
Supplement: Table S2 — Summary findings of several studies that analyzed LINE-1 global methylation in healthy human individuals. (PDF) [file pone.0016252.s006.pdf]

Supplementary Table S2:

Summary findings of several studies that analyzed LINE-1 global methylation in healthy human individuals.

	Study	Samples Characteristics				Method used	Results		Ref.
		Sample size	age	gender	tissue		age	gender	
**1**	Bollati V et al, 2009	1097 (718 subjects)	55-92	only males	peripheral blood	Pyrosequencing	no effect-borderline decrease	Not studied	33
**2**	Wihelm CS et al, 2010	465 subjects	61.7±10.4	297M 168F	peripheral blood	Pyrosequencing	no effect	Male higher	37
**3**	Jintaridth P et al, 2010	177 subjects	20-88 (Mean= 52.92±1.19)	42M 135F	mononuclear peripheral blood	COBRA	no effect	no effect	35
**4**	Chalitchagorn K et al, 2004	32 subjects	not reported	16M 16F	peripheral blood	COBRA	no effect	no effect	11
**5**	Figueredo JC et al, 2009	388 jubjects	57.8±9.1	246M 142F	Colon tissues (left and right)	Pyrosequencing	no effect	no effect	38
**6**	Iacopetta B et al, 2007	178	68.8±13.9	124M 81F	Colonic mucosa	Methylight	no effect-tendency to decrease	no effect	34
